# G. B. Fraser

**Published:** 1906-09

**Authors:** 


### G. B. FRASER, M.R.C.S., L.S.A., Weston-super-Mare.

Græme Bisdee Fraser, *æt.* 53, was the son of Peter Gordon Fraser, a former Colonial Secretary of Tasmania. He received his medical education at St. Mary's Hospital, and obtained the M.R.C.S. England in 1877, and the L.S.A. in 1878. In 1876-7 he was Prosector to the Royal College of Surgeons, England; and he held the posts of Senior House Physician and Senior House Surgeon at St. Mary's Hospital.

Soon after qualifying he settled in practice in Weston-super-Mare, where he quickly attracted a large connection. He continued in full work, and was assisting at an operation on the same day that he was seized with the pleurisy and pneumonia from which he died on August 8th, 1906. For some weeks before he had been troubled with a very severe spasmodic cough, apparently influenzal in its nature, which had given him very great distress and interfered with his sleep at night. Probably the pneumonia was of the same origin.

As a doctor he was both skilful and successful, more particularly in his treatment of the diseases of women; he possessed good judgment and sound common sense. By his patients he was esteemed and appreciated, both for his skill and for the kindly sympathy and interest he devoted to them, so that they felt that in him they had not only a clever doctor but also a sympathetic, warm-hearted friend.

Among the various appointments he held may be mentioned Medical Officer to the Royal West of England Sanatorium, and to the Convalescent Home connected with the Bristol Royal Hospital for Sick Children and Women, in which both he and Mrs. Fraser took very keen interest. For many years he was Captain of the Weston-super-Mare Fire Brigade, and also Surgeon-Colonel to the Devon and Somerset Volunteer Engineers.

From a social point of view Græme Fraser was a most popular man, wherever he went he made friends and kept them. He was always the same, with a pleasant greeting for all, and he never bore resentment.

As a boy and young man he was a first-class sprinter, a good shot, a keen fisherman, and he frequently rode to hounds. He was a singularly "handy man," and not only painted with great skill and taste, but also produced some wood-carving of exceptional merit.

The writer of this notice knew him very intimately for many years, both as a brother-doctor and as a great friend, and he will always remember him as a man of the sweetest disposition, sympathetic and affectionate in his friendship, generous and just in his dealings with his fellow-men.

